# Round the Hospitals

**Published:** 1917-12-01

**Authors:** 


### ROUND THE HOSPITALS.

The College of Nursing since the holidays has shown increasing activity, and the staff are kept busy, especially Miss Rundle, who, despite the decreased railway facilities and the added cost, is to be found on Monday in the Midlands and at Manchester towards the week-end. It is due to her, and will be welcome to all who have registered with the College to know, that the meetings she is holding are increasing the members and results for the College. The number of nurses registering with the College grows steadily, and in one case one of the Territorial sisters handed Miss Rundle a contribution for the Nation's Fund for Nurses, which constitutes the first donation from a member of the College to that Fund. It is evidence of the hold the College has gained and is gaining in public confidence that the British Press is solidly behind the British Women's Hospital in its effort to raise the Nation's Fund for Nurses to an adequate amount. This page this week illustrates the force and truth of the views just expressed. The latest most welcome help has come from the *Lancet* and the *Medical Officer*.

At Leicester on November 19 Miss Rundle, R.R.C., on the invitation of the Principal Matron, Miss Vincent, R.R.C., addressed two meetings of trained nurses at the Territorial hospitals, Leicester, on the aims and objects of the College of Nursing. The Officer Commanding, Colonel Harrison, R.A.M.C. (T.), took the chair at the base hospital of the 5th Northern General Hospital, and was supported by Major Wallace Henry, R.A.M.C. (T.), the registrar of the 1/5th Northern General Hospital. Miss Rundle's success in Leicester is demonstrated by the report that her address was most excellent and clear, and enabled those present readily to grasp why the College was founded and what it had set out to do. Another proof was afforded by the keen discussion which followed, with the number of questions that were asked and answered with telling effect. Much enthusiasm was exhibited for the College, and in the result many of the trained staff present joined the College of Nursing there and then, and paid their membership fee. The medical officers impressed upon the trained staff how important a matter it was to them that they should systematically organise themselves as a large and powerful trained body, when nurses would be in a position to compel attention to their own views and needs.

Equal enthusiasm was exhibited at the meeting at the North Evington War Hospital Extension, at which the administrator, Captain Holmes, presided. He, too, was insistent upon the importance for trained nurses to unite and organise themselves as one body through the College of Nursing, which was calculated to become a bulwark of strength to British nurses throughout the Empire if they promptly co-operated with it and closed their ranks. Miss Rundle's address was again much appreciated,

**ROUND THE HOSPITALS—(*continued*).**

and Leicester has the distinction of being the first centre of Territorial nurses which has spontaneously contributed to the Nation's Fund for Nurses. Leicester is a centre of life, energy, and efficiency, and what Leicester has done to-day other College members and Territorial sisters are likely to do cheerfully and heartily by moving their friends and contributing themselves to the Tribute Fund.

What is described as a very successful meeting was held at the Manchester Royal Infirmary on November 22, when Miss Rundle gave an interesting explanation of the aims and objects of the College and what it had been able to achieve already. The scheme of the British Women's Hospital Committee was fully explained, and the nurses present, realising that they must save for their own future, recognised too that in the ordinary course of a working life many members of the profession may fall on evil days. This made them feel what a boon a benevolent fund such as the Tribute Fund must prove to British nurses. It will give confidence and help, bringing a quiet and contented mind to the working nurse, who, Manchester felt, would take courage and set to work to organise her fellows under the College of Nursing, a sentiment which elicited warm cheers at the meeting at the Royal Infirmary, at which upwards of 200 certificated nurses were present. It is by no means easy for the secretary of a great institution like the College in its early days to address a large meeting, when the majority of those present are entire strangers, and their sentiments and views are unknown to the speaker. Manchester, which has a national character for grit and shrewdness, evidently appreciated Miss Rundle's effort, for the meeting passed what is described as a hearty vote of thanks to Miss Rundle, and those present will no doubt clinch that vote by sending in their names to be entered on the Register of the College of Nursing.

(*Continued on page* 192.)

**ROUND THE HOSPITALS**—*(continued)*.

Through the kindness of Mrs. Armstrong, matron of the Victoria Hospital, Cork, a most successful meeting on behalf of the Irish Branch of the College of Nursing was held in that hospital on Thursday, November 22. About 100 nurses were present, including Sister Albeus Fogarty, matron of the South Infirmary; Sister Angela, matron of the North Infirmary; and Miss McCreery, matron of the military hospital at Victoria Barracks. The chair was taken by Professor Ashley Cummins, who drew attention to the wide scope of the College and the necessity for unity amongst nurses. Miss Matheson, Secretary of the Irish Board, in explaining the objects of the College, pointed out the different needs of trained nurses and the way in which the College could meet these needs. An interesting informal discussion followed, in the course of which several questions were asked and answered. Sister Albeus, matron of the South Infirmary, evinced the keenest sympathetic interest in the question of training and examination subsequent to the period of grace, and expressed satisfaction that Irish nurses could take the examination of the College without going to England. Dr. Arthur Sandford apologised for the fact that, although a member of the Irish Board, he had not hitherto taken the trouble to find out much about the College, a defect which was now fully remedied. When he first heard that there was going to be a College of Nursing he was delighted, as he had always felt that something of the kind was badly needed. And he was sure that the College would be able to accomplish a great deal for the nurses and was well worthy of their support. Miss Matheson is rendering most excellent service to the nursing profession by her untiring industry and the marked ability she brings to bear in the discharge of the duties of Secretary of the Irish Board of the College of Nursing.

Miss Matheson, in her talk with nurses at Cork, said: " I have tried to show you in broad outline something of what the College of Nursing, Limited, can accomplish for us as trained nurses. I would like, with your permission, to try and see how the College affects Ireland and Irish nurses. I think all of you must see that the nursing profession is all one, though its thousands of individual units may differ from one another in everything else except the fact that they are nurses, that they love their work, and honour their profession. As far as their life apart from their profession goes, the books they read, the friends they have, the amusements they like, the church they go to, the part of the world that means home to them, the form of Government that they would choose to live under, in short, everything that interests them, in all these many of them are as far asunder from one another as Ireland is from Madagascar. But, although as individuals, and in what I might call their *mufti* life, all these things are vitally important to them; as nurses they matter not one whit. The only things that matter then are the things that affect their profession and their life as nurses. For this reason the interests of Irish nurses are identical with those of all other English-speaking nurses. And therefore the needs to which I referred in explaining the College are the needs of Irish nurses, and can only be fully met by the same organisation which, because it is and will be the strongest organisation of the profession, is going to meet the needs of English, Scotch, Welsh, and Colonial nurses. Our interests are identical, but our conditions are not always the same as those in England, and we have difficulties of our own. For this reason we have an Irish Branch of the College to deal with Irish conditions. On its Board are Irish nurses and doctors from Dublin, Belfast, Cork, Waterford, Limerick, and Galway. The Irish Board deals with nurses trained in Ireland who wish to become members of the College of Nursing, will in the future be responsible for the examinations of the College in Ireland, and will generally see that the objects of the College are carried out here, and that Irish members share all the advantages to which membership entitles them."

There is nowadays something scientific in the culinary affairs of institutions, and now the mechanic is to have his place in the kitchen. For reasons of scarcity of staff the Metropolitan Asylums Board recently decided to install mixing machinery in some of the institutional kitchens. Some of these mixers and other such appliances have now come to hand from America, where a feature is made of culinary economy, and the machines have been fixed in the institution kitchens. The moment the Asylums Board knew that the machinery was on its way it took steps to arrange for the training of men in the management of the American fittings. The Board wisely felt that the employment of a skilled man to work the whole of the machines in each kitchen would prove the most efficient means to justify a reduction in the former staff to the extent of two domestics per kitchen as a minimum. The Metropolitan Asylums Board has created a new office, that of the cook's assistant, whose duty it will be to assume control of the mechanical cooking appliances and to make himself generally useful. We may mention, too that at Tooting Bec Asylum there have been labour-saving devices in the kitchen for quite a long time. Has the plan here referred to come to the notice of any hospital managers? If so, we should welcome particulars and drawings.

☞ It is our invariable practice to pay for all items of news which we may publish. The minimum payment is 5s. Paragraphs of about twenty lines in length—that is to say, of 150 words or less—are preferred. Contributions should be addressed to The Editor, The Hospital, 28 and 29 Southampton Street, Strand, London, W.C. 2, and, to be in time for the current week's issue, should reach him by the first post on Mondays.

For Nursing Affairs in Ireland, see p. 188; Matrons' and Sisters' Appointments, see p. 194.

